# Spatial and temporal migration of sweat: from skin to clothing

**DOI:** 10.1007/s00421-018-3941-9

**Published:** 2018-07-19

**Authors:** Margherita Raccuglia, Christian Heyde, Alex Lloyd, Simon Hodder, George Havenith

**Affiliations:** 1 Environmental Ergonomics Research Centre, Loughborough Design School, Loughborough University, Loughborough, Leicestershire LE11 3TU UK; 2 Adidas FUTURE Sport Science, Herzogenaurach, Germany

**Keywords:** Sweat in clothing, Whole body sweat, Sweat mapping, Garment fit, Sportswear development

## Abstract

**Purpose:**

Moisture accumulation in clothing affects human performance and productivity through its impact on thermal balance and various aspects of discomfort. Building on our laboratory’s work on mapping sweat production across the body, this study aimed to obtain detailed spatial and temporal maps showing how this sweat migrates into a single clothing layer (T-shirt) during physical exercise.

**Method:**

Eight male participants performed running exercise in a warm environment. Garment sweat absorption was mapped over a total running time of 50 min, in 10 separated running trials of different durations (5 min increments). After running, the garment was dissected into 22 different parts and local sweat absorption (ABS_local_) was quantified by weighing each garment part before and after drying. From ABS_local_, garment total sweat absorption (ABS_total_) was estimated.

**Results:**

After 50 min, *T*_core_ rose from 37 ± 0.2 to 38.6 ± 0.3 °C, HR increased from 69 ± 15 to 163 ± 12 bpm (*p* < 0.001), GSL was 586 ± 86 g m^−2^. Clear patterns of sweat absorption reduction from superior-to-inferior and from medial-to-lateral T-shirt zones were observed, with the mid back medial and the low front hem showing the highest, respectively.

**Conclusions:**

Quantitative data on garment total and regional sweat absorption were obtained and considerable variation between different garment zones was identified. These data can support the development of sport and personal protective clothing with the end goal to prevent workers’ heat-related injuries as well as maximise human performance and productivity.

**Electronic supplementary material:**

The online version of this article (10.1007/s00421-018-3941-9) contains supplementary material, which is available to authorized users.

## Introduction

Humans wear clothing as means of embellishment, status and modesty. More importantly for human health and survival, clothing provides the body with a protective physical barrier from environmental factors, such as rain, snow, wind and solar radiation. Beside this imperative protective function, the interaction between clothing and the human body has implications in terms of biophysics of heat transfer, temperature regulation and comfort (Havenith 1999; Morrissey and Rossi 2013; Jay and Brotherhood 2016). When exposed to hot environments, sweat evaporation occurs to maintain body thermal balance, representing the greatest avenue for body heat loss in exercise (Candas et al. 1979). However, the clothing barrier impairs evaporative heat loss from the body, this in part causing less efficient sweat evaporation (Candas et al. 1979; Shapiro et al. 1982; Havenith et al. 2007, 2013). Once body heat production or the evaporative resistance of clothing increases to the point where sweat evaporation cannot keep up with sweat rate, the skin becomes saturated with sweat and the garment worn will also get wet. In the cold and/or when metabolic heat production is reduced right after physical exercise, skin and clothing wetness cause a fast decrease in body temperature (Li 2005). In this scenario, both skin and clothing wetness can lead to thermal discomfort, cold sensations and, in extreme conditions, to hypothermia.

Apart from its impact on body heat loss, the presence of wetness also exacerbates the tactile interaction between the skin and the fabric, sensed by the wearer as stickiness (Filingeri et al. 2015; Raccuglia et al. 2017). Hence, wetness represents one of the most important sources of discomfort when wearing clothing, (Hong et al. 1988; Raccuglia et al. 2016) which could even contribute to decrements in human performance and productivity (Parsons 2014; DenHartog and Koerhuis 2017).

Extensive research has been conducted to discover strategies able to maximise heat and mass transfer through the clothing barrier, yet maintaining its protective function (Lomax 2007; Fukazawa and Havenith 2009; Sarkar et al. 2009; Havenith et al. 2011; Ke et al. 2013; Sun et al. 2015; Lin et al. 2015; Wang et al. 2017). To characterise fabric moisture absorption and transport properties, several apparatus and test methods have been developed (Harnett and Mehta 1984; Hong et al. 1988; Ghali et al. 1994; McCullough et al. 2003; Huang and Qian 2007; Huang and Xiaoming 2008); however, the relevance of these tests has not been supported by real-life data from humans during exercise. Previous studies have provided data of regional sweating rates in humans during rest and exercise (Cotter et al. 1995; Taylor et al. 2006; Machado-Moreira et al. 2008a, b, c; Smith and Havenith 2011, 2012). These data made available fundamental knowledge on sweat rate patterns across the human body that might support the process of sportswear and protective clothing development. Nevertheless, it is unknown how the complex body shapes, draping of clothing, air gap and contact area (Psikuta et al. 2012; Frackiewicz-Kaczmarek et al. 2015) between the garment and the body impact sweat absorption values and patterns in clothing. In fact, wicking properties of clothing are not only determined by the amount of sweat produced at specific body locations, but can also be affected by the thickness of the air gap and the contact area between the garment and the human body (Psikuta et al. 2012; Frackiewicz-Kaczmarek et al. 2015). These parameters can be easily defined for a tight-fitting clothing item, as the sweat transfer between skin and such a garment would be expected to be similar to the body sweat pattern, given that these patterns were produced using absorbent material directly in contact with the skin (Havenith et al. 2008; Smith and Havenith 2011, 2012). Therefore, the aim of the current study was to provide detailed maps of sweat accumulation across a regular-fitting upper body garment, induced in male athletes during running exercise. The use of a regular-fitted garment, as the most commonly used fit, allows determining the impact of clothing and personal factors on garment sweat absorption and migration.

Realistic sweat absorption data can support the development of garments with efficient moisture management features, e.g., with spatial variation of textile types. In terms of real-world impact, improvements in clothing moisture management can lead to improvements in heat loss efficiency as well as reductions in discomfort. A sensation of lower discomfort can boost people’s willingness to be physically active, thereby having a beneficial effect on health and well-being, along with improving performance and productivity of athletes and workers (Parsons 2014; DenHartog and Koerhuis 2017). Finally, the current findings can advance the existing knowledge on thermophysiological modelling.

## Materials and methods

### Participants

Eight male, long-distance runners were recruited from the Loughborough University student cohort. The mean (± standard deviation) age was 23.3 ± 4.7 years and they were all of Western European origin. Their body mass, height and body fat was 70.0 ± 9.9 kg, 177.3 ± 5.3 cm and 9.6 ± 4.5%, respectively. They were all training six times per week and the mean aerobic fitness level, measured as maximum oxygen uptake (*V*O_2max_), was 62.0 ± 3.0 mL kg^−1^ min^−1^. Immediately after the completion of the current study, four of the eight participants repeated the 10 experimental trials wearing a synthetic garment (sub-study). With regard to these four participants, the mean age was 20.3 ± 2.9 years. Their body mass, height and body fat was 66.8 ± 12.3 kg, 175.6 ± 7.0 cm and 8.6 ± 3.7%, respectively. Their *V*O_2max_ and running speed was 62.1 ± 3.7 mL kg^−1^ min^−1^ and 12.3 ± 0.6 km h^−1^.

The experimental procedures were fully explained to the participants verbally and through written information form, before obtaining written informed consent and completing a health screening questionnaire. All the experimental procedures involved were approved by the Loughborough University Ethical Committee. The study was conducted within the confines of the World Medical Association Declaration of Helsinki for medical research involving human participants.

### Pre-test

Participants were required to attend the laboratory for a pre-test, involving anthropometric measurements of height, body mass (Mettler Toledo Kcc150, Mettler Toledo, Leicester, UK), percentage of body fat (Tanita Corporation, Tokyo, Japan) and body dimensions.

During the pre-test, participants also performed a sub-maximal fitness test to estimate their aerobic fitness level, expressed as maximal oxygen uptake (*V*O_2max_). The sub-maximal fitness test was performed according to the American College of Sport Medicine guidelines for exercise testing and prescription (Thompson et al. 2010). The sub-maximal fitness test was conducted at an ambient temperature of 20 °C and 50% relative humidity. The test comprises a series of running stages on a treadmill (h/p/cosmos mercury 4.0 h/p/cosmos sport & medical gmbh, Nussdorf-Traunstein, Germany). A system for the measurement of oxygen consumption (*V*O_2_), including heart rate (HR) (COSMED Quark CPET Series, COSMED, Srl, Italy) was used. During the test, the exercise intensity was progressively increased by changing the running speed by 2 km h^−1^ every 4 min. The treadmill incline was not altered and maintained at 1% for the entire duration of the test. Each stage lasted 4 min to ensure a steady-state HR response. The end point of the test was determined when participants reached 85% of the individual age-predicted maximal HR (220—age) (no more than five stages were performed). As long as work intensity is increased adequately (equal speed increment and duration of each stage) a linear relation can be observed between HR and *V*O_2_ measured at the end of each running stage, and based on this relation, *V*O_2max_ was estimated from the age-predicted maximal HR. A similar linear relation can be observed between running speed and VO_2_ at each running stage, and this was used to establish the corresponding speed for the testing (70% *V*O_2max_).

### Experimental conditions

**Fig. 1:**
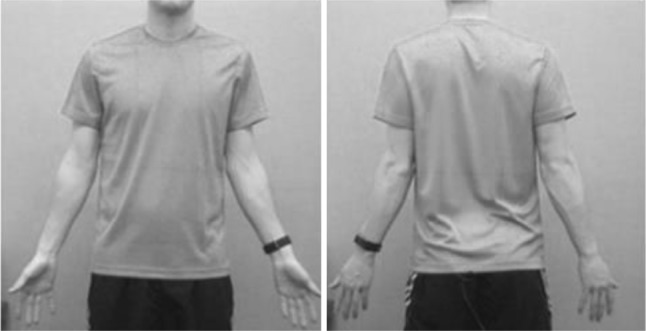
Anterior and posterior picture of a participant wearing an experimental garment, here defined as regular-fitted

Sweat absorption across the T-shirt was mapped at 5-min intervals over a total running time of 50 min. As a ‘destructive’ gravimetric method was adopted to quantify regional sweat absorption, each participant performed 10 different running trials on a treadmill, characterised by different durations: 5, 10, 15, 20, 25, 30, 35, 40, 45 and 50 min. In fact, immediately after each running trial, the T-shirt was dissected into different parts (Fig. 1) and each part was analysed to determine the time course and distribution of sweat absorption over the duration of each run duration. In all the trials, the participants ran at the same individually fixed speed, corresponding to 70% of *V*O_2max_; the mean running speed was 12.1 ± 0.7 km h^−1^. The experiment was conducted in a small wind tunnel located in a climate-controlled chamber maintained at 27.2 ± 0.2 °C, 49.7 ± 3.2% relative humidity and 1.5 m s^−1^ wind speed. These specific environmental parameters were applied to allow direct comparisons with previous studies investigating body regional sweat rate patterns (Smith and Havenith 2011, 2012).

### T-shirt specification

A fresh pre-washed (ISO 6330 2012), regular-fitted, short sleeved, T-shirt was used for each of the 10 run durations. Sweat absorption and distribution was mapped in a 100% cotton garment, which, due to the higher hygroscopicity and greater capacity to retain liquid moisture, would represent the most challenging scenario for textile and clothing developers. The synthetic garment (100% polyester), included in the sub-study, was characterised by different thermal, evaporative and wicking properties from the cotton garment. The aim of this sub-investigation was to demonstrate that the properties of the fabric can affect sweat absorption values, rather than simply highlight differences between a natural and a synthetic garment. The synthetic garment was produced using the same fit pattern of the cotton garment. Material specifications of the cotton and synthetic garments are in Table 1.

**Table 1:** Specifications of the experimental garments

Garment	Mass (g m^2^)	Thickness (mm)	*R*_ct_ (m^2^ °C/W)	*R*_ef_ (m^2^Pa/W)	Air perm (mm s^−1^)	Absorption (g m^−2^)
100% cotton	159	0.55	0.02	3.1	780	381
100% polyester	127	0.46	0.01	2.2	2088	368

Dry thermal resistance and water vapour resistance were measured according to BS EN ISO 11092:2014, air permeability was measured according to BS EN ISO 9137; total absorption capacity was measured according to the absorption capacity test adopted by Raccuglia et al. (2016), modified from Tang et al. (2014)

*R*_ct_ dry thermal resistance, *R*_*ef*_ water vapour resistance, *Air perm* air permeability, *Absorption* total absorption capacity

To better describe the fit design of the garment, here defined as ‘regular’, anterior and posterior picture of a participant wearing an experimental garment are provided in Fig. 1.

To ensure same regular fit between participants presenting different body dimensions, three different T-shirt sizes were included (Small, Medium and Large). The waist circumference of the participants was measured horizontally at level of the waist (where the smallest abdominal circumference occurs), while the person stands erect with the arms held slightly away from the side of the body. Three ranges of waist circumference were identified, small (68–73 cm), medium (74–79 cm) and large (80–85 cm). The circumference of each garment size, measured at the waist circumference of the participants, was taken. The latter was 90 cm for the small size, 100 cm the medium size, and 110 cm for the large size, used for small (68–73 cm), medium (74–79 cm) and large (80–85 cm) waist circumference range, respectively.

**Fig. 2:**
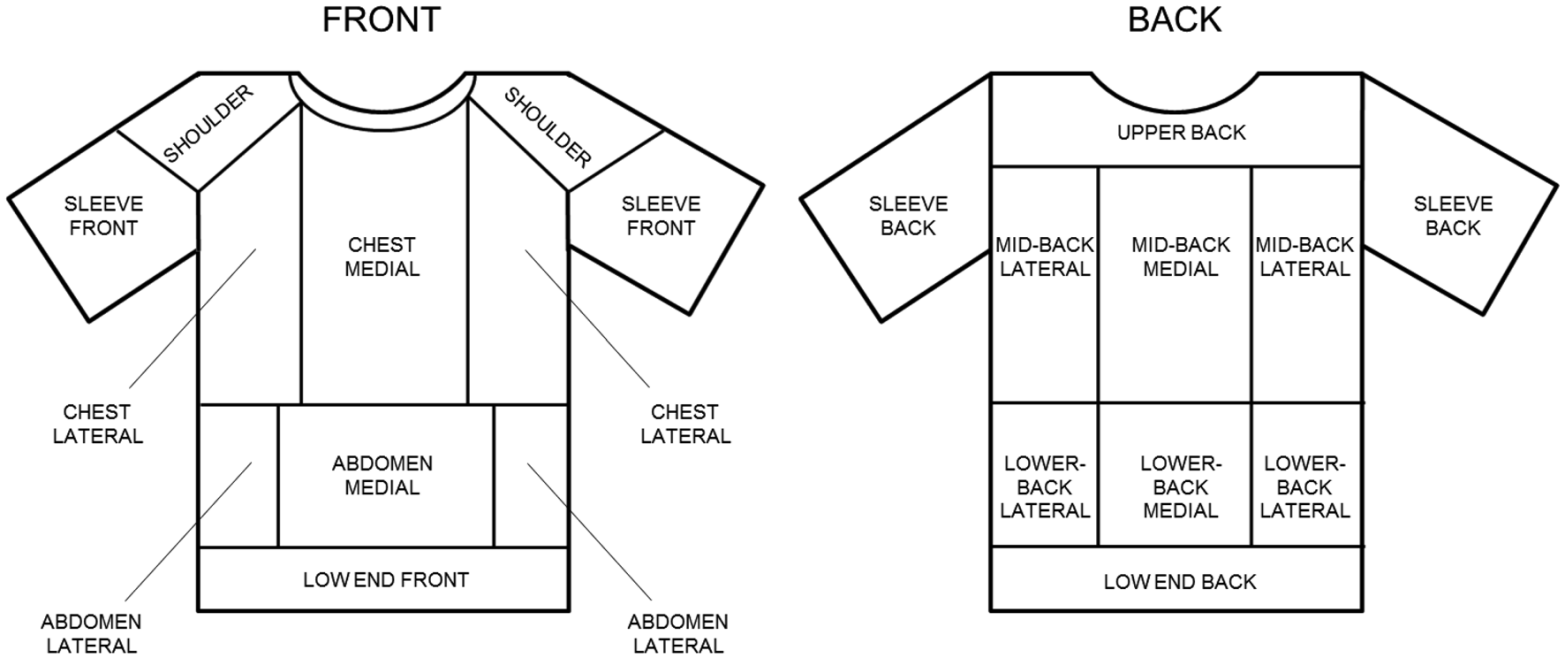
Schematic representation of the experimental T-shirt marked into the 22 regions of interest for the analyses of local sweat accumulation. Front and back of the T-shirt were mapped into 12 and 10 zones, respectively

**Fig. 3:**
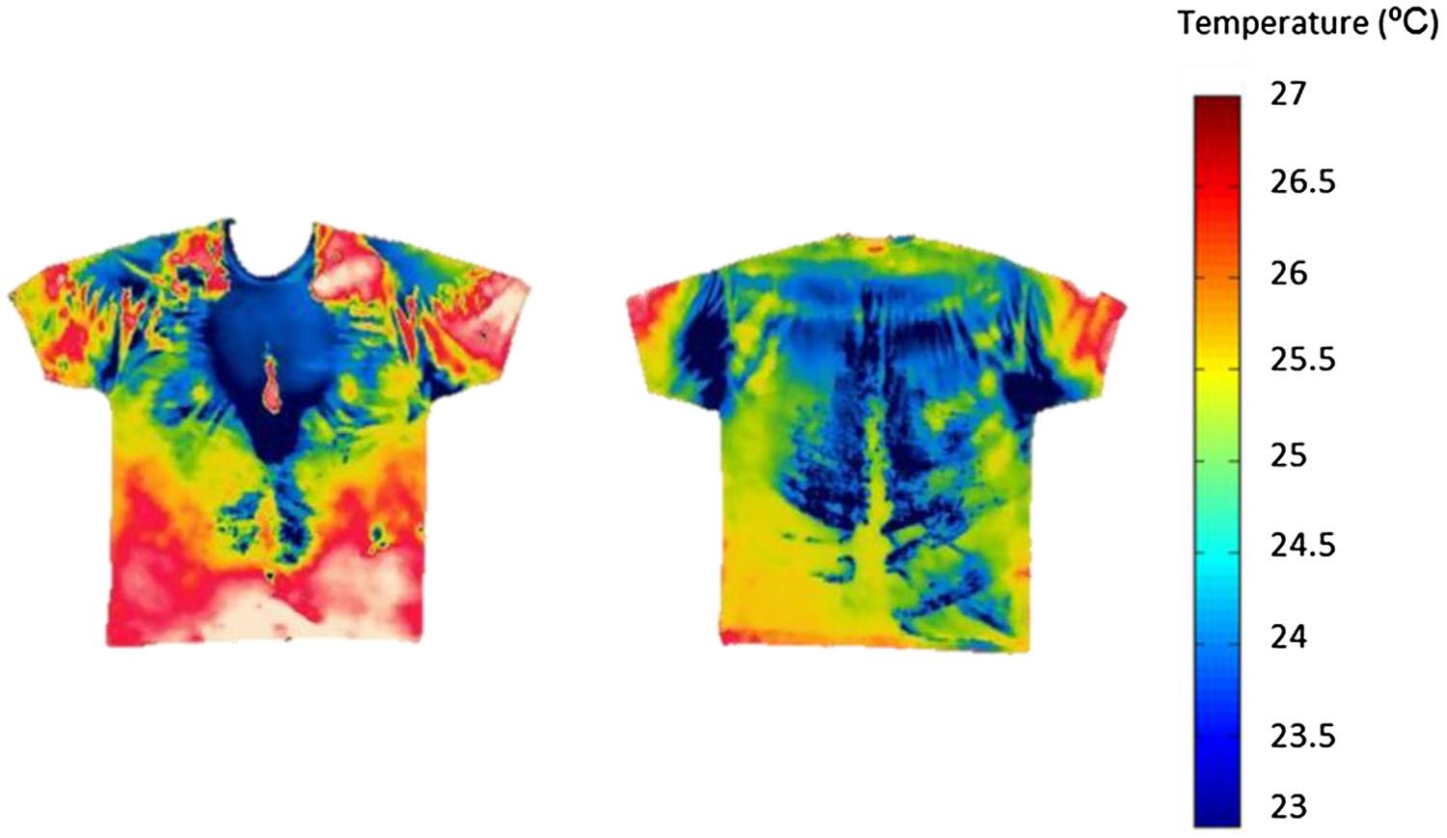
Example of infrared pictures of front and back of the T-shirt after 30 min of running exercise taken to identify variations in sweat retentions between various T-shirt regions

Analyses of T-shirt local sweat absorption (ABS_local_) were conducted in 22 regions of the T-shirt, 12 for the front and 10 for the back, respectively (Fig. 2). The relevant sweat absorption zones within the T-shirt were selected based on temperature patterns highlighted in infrared pictures (conducted in pilot testing), taken once the T-shirt was taken off (Fig. 3). At the end of each run duration, analyses of local sweat absorption were conducted by cutting up the marked T-shirt regions and weighing the individual sections before and after drying.

### Experimental protocol

Participants performed the 10 running trials on different days, separated by at least 24 h of rest. Including the pre-test, each participant performed 11 visits in total and undertook only one running trial per day. The testing sequence was counterbalanced to prevent any order effect. Each participant performed the trials at the same time of the day to minimise circadian variation. Participants were instructed to refrain from strenuous exercise, abstain from caffeine and alcohol consumption 24 h before testing, and to keep a record of their food intake and replicate it the day before each visit. To maintain euhydration, they were also advised to consume 20 mL kg^−1^ body weight of water during the 2 h prior to testing. On arrival to the laboratory participants were asked to void their bladders, self-insert a rectal probe, for the measurement of core body temperature, and wear a wrist-based HR monitor. Following from this, semi-nude (including underwear, rectal probe and HR monitor) body mass was recorded. Subsequently, participants were provided with standard running shorts and socks, wore their personal running shoes. Participants were asked to use the same personal running gears for the entire duration of the experiment. This period of preparation lasted approximately 15 min and allowed time for the stabilisation of HR and *T*_core_. Participants moved to the climate-controlled room, rested standing still on the treadmill and after 10 min baseline HR was recorded. They, then wore the experimental T-shirt and the running trial started. To prevent dehydration, the participants were allowed to drink water ad libitum during the experiment, and liquid consumption was recorded. At the end of the run participants took the worn T-shirt off which was given to the experimenter for measurement of local sweat absorption. The participants took shorts, sock and shoes, towelled their skin (this took ~ 2 min) and post-exercise semi-nude body mass was recorded immediately.

### Measurements

Heart rate was recorded before (baseline, BL) and during the running trials at 1 min intervals with a wrist-based heart rate monitor (Polar A360, Polar Electro Oy, Professorintie 5, Kempele, Finland). A wrist-based monitor, rather than a chest-based monitor, was used since a chest strap would have interfered with sweat transfer from the skin to the T-shirt. To monitor changes in *T*_core,_ rectal temperature was recorded via a rectal thermistor (Grant Instrument Ltd, Cambridge, UK), inserted 10 cm beyond the anal sphincter. Rectal temperature was measured throughout each experimental trial at 1-min intervals and recorded via a portable data logger (Grant Instrument Ltd, Cambridge, UK) connected to the thermistor probe. Sweat production was calculated based on the weight change of each participant (gross sweat loss, GSL), corrected for liquid intake, and reported in grams per body surface area (g m^−2^), according to:

$${\text{GSL }}\left( {{\text{g}}~{{\text{m}}^{ - {\text{2}}}}} \right)~=~[{w_{{\text{b1}}}} - {\text{ }}\left( {{w_{{\text{b2}}}} - {\text{ liquid}}} \right)]/{\text{SA,}}$$

where *w*_b1_ is the body mass at the start of the experiment (g), *w*_b2_ is the body mass at the end of the experiment (g), liquid is the total water consumption (g), and SA is the body surface area (m^2^).

### Total and local T-shirt sweat content

Extensive pilot testing was conducted to define the exact locations and the number of zones to map within the T-shirt. Two participants conducted a full set of pilot trials and at the end of each pilot test infrared picture of the T-shirt were taken to identify variations in sweat absorption between regions and over time. The infrared pictures permitted to visually detect, based on colour differences, T-shirt regions characterised by diverse temperatures. It was assumed that variations in temperature across the T-shirt corresponded to variations in sweat content, and based on this principle, the T-shirt was mapped in 22 different zones: 12 for the front and 10 for the back (Fig. 2). To the knowledge of the authors, currently there are no standardised methods able to directly and accurately measure liquid moisture content in specific clothing sections, without dissecting the garment. A gravimetric method, based on weight changes (difference between wet and dry garment weight) is typically adopted to estimate sweat absorption in a full garment. However, since each individual section of the T-shirt to be measured could not be weighed prior testing, it was decided to adopt a ‘destructive’ gravimetric method, in which each T-shirt region was cut up immediately following sweat collection, and based on weight changes local sweat content was estimated. Twelve hours before being worn, the T-shirt was marked with a permanent pen into the 22 sections and left in a climate-controlled room (20 °C, 60% relative humidity). Immediately after being taken off, the T-shirt was fitted to a T-shirt-shape wooden stand and divided into front and back panel, to prevent sweat transfer from the front to the back and vice versa. Front and back of the T-shirt were separately laid flat on a table and each pre-marked section was cut up. The order of cutting was balanced to prevent any order-related error. It took a maximum of 7 min to cut up the full T-shirt, as it was assessed in a pilot test that after 7 min, the weight of the full T-shirt starts to change due to drying. Immediately after being cut, the specific T-shirt regions were placed in individually labelled airtight bags, to prevent sweat evaporation. The weight of each wet T-shirt section inserted into the corresponding bag was recorded using a calibrated electronic weighing scale (PSK 360-3, Kern,UK), with a maximum load of 360 g and a precision of 0.001 g. After being cut, the sections were then taken off the bag and placed in a chamber at 30 °C and 7% relative humidity for 12 h, to allow the material to dry. The dried sections where then re-weighted without the bags to establish the dry weight. Local sweat absorption (ABS_local_) was calculated from the weight change and the surface area of each section according to:

$${\text{AB}}{{\text{S}}_{{\text{local}}}}\left( {{\text{g}}~{{\text{m}}^{ - {\text{2}}}}} \right)~=~[({w_{{\text{wet}}}} - {\text{ bag}}){\text{ }} - {w_{{\text{dry}}}}]/{\text{SA,}}$$

where *w*_wet_ is the section wet weight, including bag (g), *w*_dry_ is the section dry weight (g), bag is the mass of the airtight bag (g), SA is the section surface area (m^2^).

To calculate the SA of each T-shirt section, five control samples of the T-shirt’s fabric were produced and the dry weight per unit area (g m^−2^) was calculated from size and weight of each control sample according to:

$${\text{ASW }}\left( {{\text{g}}~{{\text{m}}^{ - {\text{2}}}}} \right)~=~({w_{\text{c}}}/{a_{\text{c}}}){\text{ 1}}0,000,$$

where ASW is the Area specific weight, *w*_c_ is the weight of control material (g), *a*_c_ is the area of control material (cm^2^).

The area weight of the control samples was stable, showing only 1.3% coefficient of variation. The mean value of the calculated weight per unit area of the five control samples was used in the calculation of each T-shirt section SA in g m^−2^ according to:

$${\text{SA}}~=~{w_{\text{d}}}/{\text{ASW,}}$$

where *w*_d_ is the dry weight of material (g).

Since weight measurements of the wet full T-shirt after the sweat collection period could have caused sweat transfer through contact between T-shirts regions, ABS_total_ was calculated from the sum of ABS_local_ according to:

$${\text{AB}}{{\text{S}}_{{\text{total}}}}~=~\left( {\sum {{\text{AB}}{{\text{S}}_{{\text{local}}}}} } \right),$$

where ABS_local_ is the sweat accumulated in a specific T-shirt region (g).

### Statistical analysis

Differences in HR and *T*_core_ recorded at same time points in different run durations were assessed with paired *t* test (2 comparisons) and one-way repeated measures ANOVA (more than 2 comparisons). HR and *T*_core_ data were averaged across same time points, and one single mean value per time point is reported and displayed in the figures.

One-way repeated measures ANOVA tests were performed to assess differences in and HR, *T*_core,_ GSL and ABS_total_ between run durations. When statistical differences were observed, post hoc tests with Bonferroni correction for multiple comparisons were conducted. As the progressive development of GSL and ABS_total_ was measured in different trials, the data were combined and reported over time.

Local sweat absorption data (ABS_local_) were first analysed to assess differences in corresponding right–left zones (shoulders, sleeves front, sleeves back, chest lateral, abdomen lateral, lateral mid-back, lateral lower-back). Paired *t* tests were performed for all the right–left zones with Bonferroni correction for multiple comparisons. To assess differences between local sweat absorption data, one-way repeated measures ANOVA tests were conducted. The large number of comparisons between zones can cause inflation of the type I error; nevertheless the application of Bonferroni correction to adjust for multiple comparisons can inflate the type II error. Therefore, it was decided to report both corrected and uncorrected *p* values, keeping in mind the exploratory nature of the research, yet recognising the conservative nature of Bonferroni correction (Smith and Havenith 2012) (Supplemental digital content).

Descriptive statistics reporting, min and max values, median, mean and standard deviation in ABS_local_ for each region was conducted. Linear regression analyses were performed to observe relations between variables, in particular between ABS_total_ and GSL. In all analyses, *p* < 0.05 was used to establish significant differences. Data are reported as mean [standard deviation (SD)]. Statistical analysis was performed using the software IBM SPSS Statistics version 23 (IBM, Chicago, USA).

## Results

There were no evident differences between the cotton and synthetic conditions in physiological data of *T*_core_, HR and GSL, obtained from the four participants taking part in both conditions. The following results refer to physiological data achieved in the experiment involving the use of the cotton garment.

### Heart rate and core temperature

**Fig. 4:**
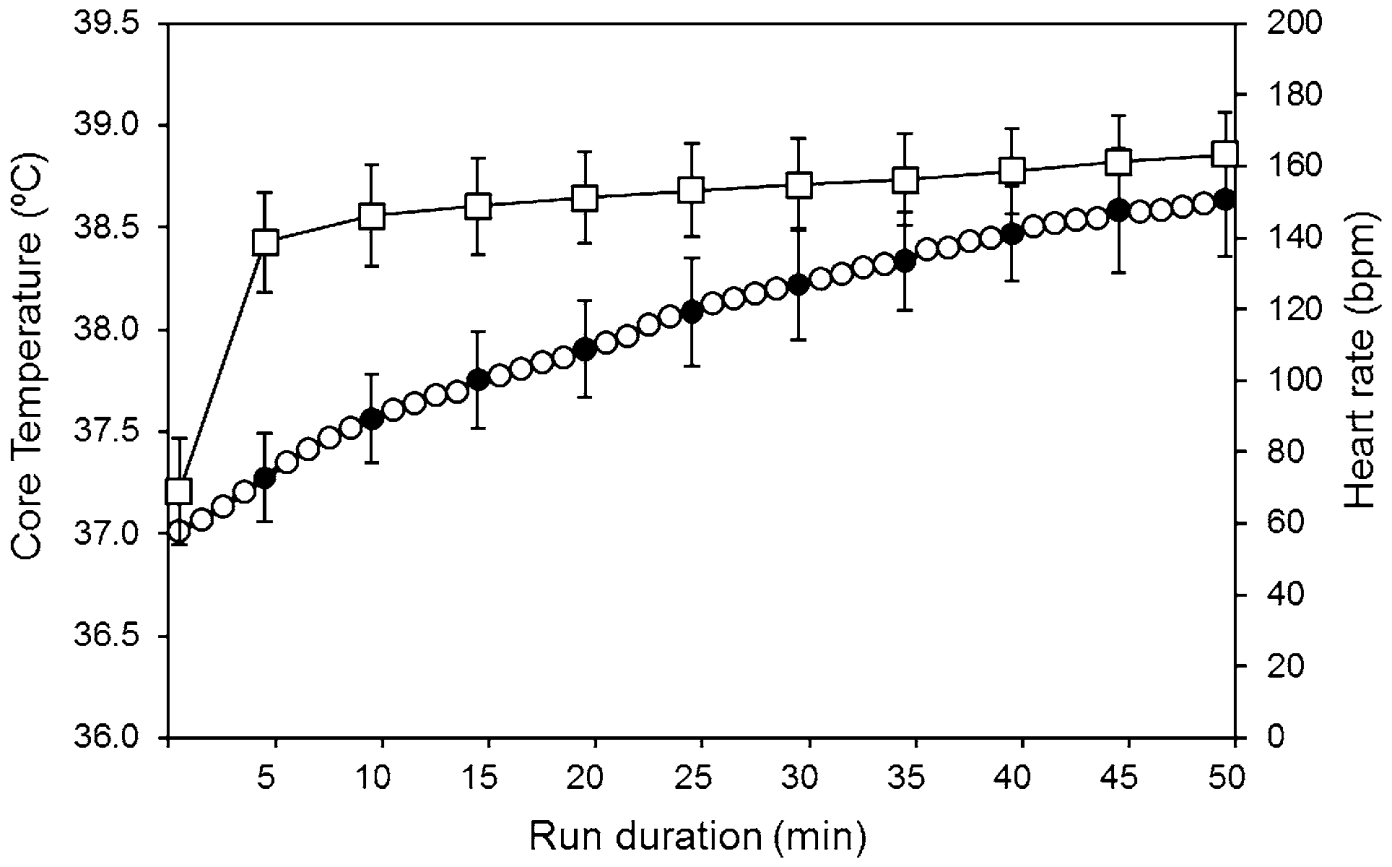
Mean core temperature (*T*_core_; circle symbols) and mean heart rate (HR; square symbols) data for 8 male athletes. Data were averaged over the 10 run durations (from 5 to 50 min). *T*_core_ and HR values were sampled at 1-min intervals. The average over 5 min is presented for HR measurements. Data are presented as mean (SD)

Heart rate (HR) and core temperature (*T*_core_) were measured at 1-min intervals throughout each running trial. Both HR and *T*_core_ were not significantly different (*p* > 0.05) at same time points and between trials, therefore data were averaged across the 10 run durations. HR and *T*_core_ both increased significantly during exercise (*p* < 0.001). Baseline HR was 69 ± 15 bpm and increased up to 163 ± 17 bpm at the end of the 50 min run duration, while *T*_core_ rose from 37.0 ± 0.17 °C, at baseline, to 38.6 ± 0.28 °C at the end of the 50 min (Fig. 4).

### Gross sweat loss

**Fig. 5:**
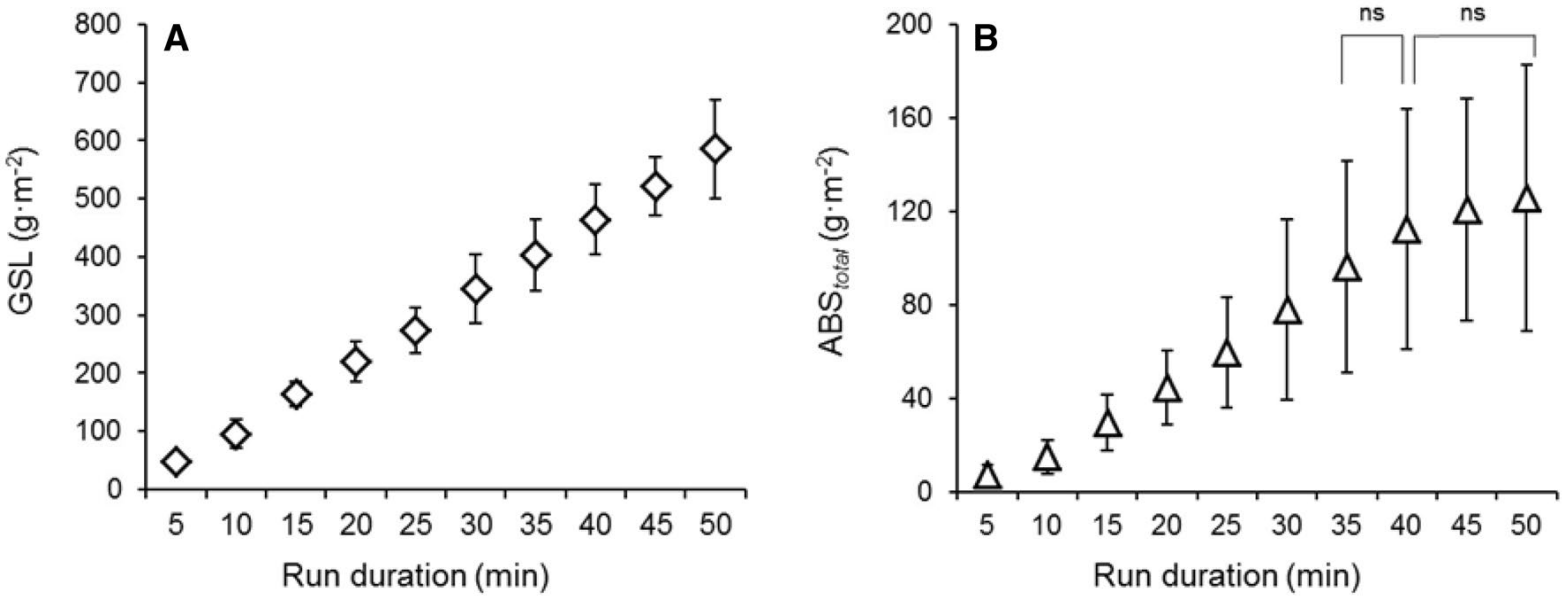
Gross sweat loss (GSL) data (**a**) and T-shirt total sweat absorption (ABS_total_) data (**b**). The mean (SD) values for 8 male athletes are presented. GSL and ABS_total_ data for each time point were obtained from 10 different run durations. GSL was significantly different between the 10 run durations. ABS_total_ was significantly different between the run durations but the differences were not significant (ns) between 35–40 and 40–45–50 min

Substantial variation in the amount of whole body produced sweat, measured as gross sweat loss (GSL), was observed between individuals (Fig. 5a). GSL was corrected for the individual body surface area (g m^−2^). Cumulative GSL linearly increased as function of run duration: at 5 min run it was 48 ± 13 g m^−2^ and the highest value was 586 ± 85 g m^−2^ observed at 50 min (Fig. 5a). The mean rate of GSL increase was 11.0 ± 0.4 g m^−2^ min^−1^.

The ANOVA test showed significant differences (*p* < 0.001) in GSL between run durations; however, when the Bonferroni correction for multiple comparison was applied, the differences were not significant (*p* > 0.05) between 30 and 35 min neither between 35 and 40 min.

### Total and local T-shirt sweat absorption

Both total T-shirt sweat absorption (ABS_total_) and ABS_local_ data between run durations were corrected for fabric surface area and reported as g m^−2^. Considerable variation in ABS_total_ was observed between individuals (Fig. 5b). ABS_total_ was greatly influenced by the large variation in GSL, indicated by a linear positive relation between ABS_total_ and GSL (*r*^2^ = 0.74, *p* < 0.001). ABS_total_ increased with the increase in run duration. The highest mean ABS_total_ value was 126 ± 57 g m^−2^ observed in the 50 min run and the mean rate of increase was 3.5 ± 0.8 g m^−2^ min^−1^. At the end of the 50 min run the T-shirt was 41% saturated, calculated as percentage of total T-shirt absorption capacity (305.5 g). This also means that, after 50 min run, 9.3% of the whole body produced sweat was collected and retained by the T-shirt (this when using absolute mean values of GSL and ABS_total,_ 1083 and 101 g, respectively).

The ANOVA test showed significant differences (*p* < 0.001) in ABS_total_ between run durations; however, the differences were not significant (*p* > 0.05) between 35 and 40 min neither between 40, 45 and 50 min.

Right and left corresponding T-Shirt regions (shoulders, front sleeves, back sleeves, lateral chest, later abdomen, lateral mid-back, lateral lower-back) did not show significant differences (*p* > 0.05) in ABS_local_ and thus left–right data were grouped for all analyses. For practical reasons, descriptive statistics for all the regions are reported only for 10, 20, 30, 40 and 50 min run durations (Table 2).

**Table 2:** Descriptive statistics of sweat absorption data for all the T-shirt (cotton) regions of interest. Reported data are in g m^−2^

	Area	10 min					20 min					30 min					40 min					50 min				
	m^2^	Min	Max	Median	Mean	SD	Min	Max	Median	Mean	SD	Min	Max	Median	Mean	SD	Min	Max	Median	Mean	SD	Min	Max	Median	Mean	SD
Collar	0.017	0	133	14	40	49	33	208	113	115	68	28	239	129	146	79	53	251	176	177	67	53	318	174	171	93
Shoulders	0.032	2	25	8	10	7	15	52	27	29	14	21	102	42	50	30	35	148	108	*95*	41	32	173	104	103	58
Sleeves front	0.030	5	20	9	10	5	5	48	18	21	13	14	61	21	31	19	7	89	32	43	28	13	133	41	57	44
Chest medial	0.053	7	57	11	18	18	19	206	75	101	72	25	258	135	139	92	55	250	191	172	82	44	245	203	171	83
Chest lateral	0.040	5	15	9	9	4	6	83	15	31	28	17	132	37	55	46	17	200	106	106	79	26	242	105	115	82
Abdomen medial	0.043	4	21	10	10	5	6	52	17	23	17	9	258	24	68	90	6	257	115	122	106	7	275	97	117	104
Abdomen lateral	0 029	1	7	6	5	2	5	13	10	9	2	5	60	11	16	18	4	77	17	26	26	4	207	13	45	70
Low end front	0.062	0	9	5	5	2	4	99	6	17	33	5	10	7	7	1	3	12	5	7	3	4	62	23	24	20
Upper back	0.067	0	53	16	23	20	44	121	78	75	27	70	222	124	128	58	120	231	166	174	44	64	228	178	162	59
Sleeve back	0.037	7	35	19	19	9	15	69	43	41	16	31	122	55	66	30	38	138	76	89	40	51	195	89	102	47
Medial mid-back	0.048	10	87	23	35	29	46	184	125	124	40	69	265	195	181	78	119	271	223	209	52	134	271	230	215	50
Lateral mid-back	0.043	10	34	15	18	8	18	82	46	52	26	27	172	103	98	52	51	236	132	142	71	51	254	163	161	72
Medial lower-back	0.036	7	62	16	22	18	19	156	51	65	42	8	89	29	44	33	88	277	168	177	70	117	290	187	198	63
Lateral lower-back	0.032	1	23	8	9	7	8	51	23	23	14	8	89	29	44	33	9	200	54	77	68	18	235	66	94	86
Low end back	0.061	2	7	5	5	1	3	10	6	6	2	6	12	9	9	2	4	123	7	23	41	3	243	9	49	82

T−shirt local sweat absorption data of 8 male athletes are reported for 10, 20, 30, 40 and 50 min run durations Minimum (min) and maximum (max) values, along with median, mean and standard deviation (SD) is reported for each region of interest

**Fig. 6:**
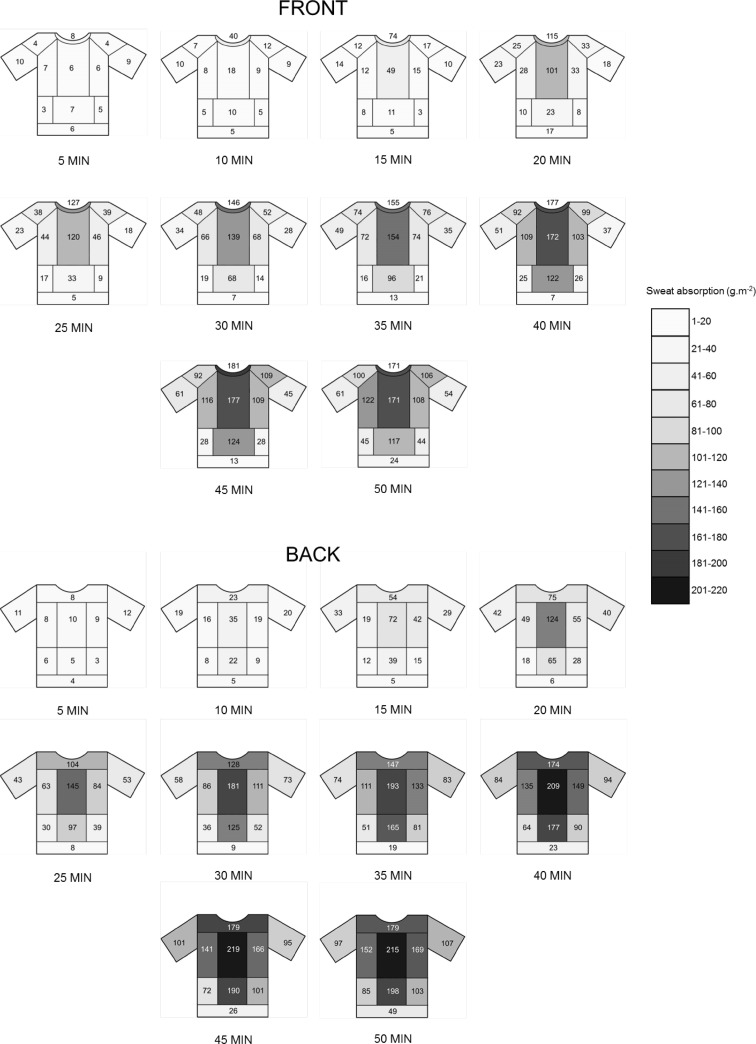
Mean T-shirt (cotton) local sweat absorption data for 8 male athletes. Local sweat absorption was measured at 5-min intervals from 5 to 50 min of running exercise, for front and back T-shirt zones. Data were obtained from 10 different run durations

Mean ABS_local_ data for front and back of the T-shirt, from 5 to 50 min, are presented in Fig. 6. Additionally, comparisons between T-shirt regions within each run condition were conducted, (Extended data set).

Local T-shirt saturation was also calculated as percentage of the total absorption capacity of the material (g m^−2^). At the end of the 50 min, medial mid-back and medial lower-back were the most saturated T-shirt parts: 56 and 51%, respectively. These were followed by upper back, collar and chest medial (40–45%), and next to these, lateral mid-back, lateral chest and lateral abdomen reached between 30 and 39% of the saturation. Shoulders, sleeves front and back and lateral lower-back were 20–29% saturated and the lowest saturation level was shown by front and back low ends together with lateral abdomen (7–12%).

A clear large variation in ABS_local_ between participants was evident from the minimum and maximum value and standard deviation data within each region (Table 1). For most of the regions mean and median values were very close, indicating normal distributions. When large differences occur, usually the mean is higher than the median, normally due to a one or two ‘high sweaters’ in the group (Havenith et al. 2008; Smith and Havenith 2011, 2012).

The coefficient of variation (CV %) in ABS_local_ between participants was calculated, to compare the variation in ABS_local_ between T-shirt regions. To achieve an overall identification of the regions with ‘higher’ and ‘lower’ variation, CV data for each region of interest were averaged over run durations. Differences in CV were observed, with hem at the back showing the highest value (99%) and the upper back the lowest (44%). The variation was higher in the inferior T-shirt regions compared to the superior ones and in the peripheral parts as compared to the central regions. When using ABS_local_ values normalised for whole individual body sweat production (ABS_local_/GSL), the CV in ABS_local_ appears to be lower (~ 6%) for all the T-shirt regions compared to the CV values obtained when using absolute data. Nevertheless, the pattern of ABS_local_ variation across T-shirt regions is similar between absolute and normalised data.

### Sub-study: synthetic garment

**Fig. 7:**
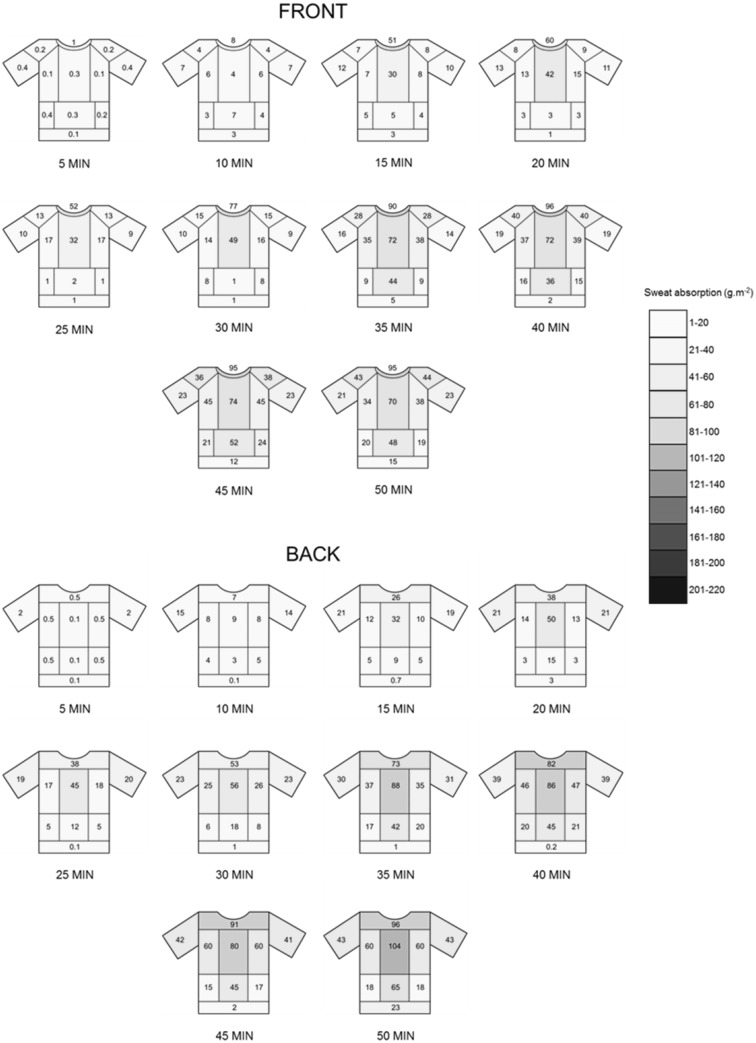
Mean T-shirt (synthetic) local sweat absorption data for 4 male athletes. Local sweat absorption was measured at 5-min intervals from 5 to 50 min of running exercise, for front and back T-shirt zones. Data were obtained from 10 different run durations

Baseline HR was 66 ± 12 bpm and increased up to 163 ± 17 bpm at the end of the 50 min run duration, while *T*_core_ rose from 37.0 ± 0.13 °C, at baseline, to 38.6 ± 0.46 °C at the end of the 50 min. ABS_total_ in the synthetic garment increased with the increase in run duration. The highest mean ABS_total_ value was 51.4 ± 27.5 g m^−2^ observed in the 50 min run. At the end of the 50 min run the T-shirt was 17.5% saturated. Mean ABS_local_ data for front and back of the T-shirt, from 5 to 50 min, are presented in Fig. 7. Descriptive statistics for all the regions are reported only for 10, 20, 30, 40 and 50 min run durations which is reported in Table 3.

**Table 3:** Descriptive statistics of sweat absorption data for all T−shirt (synthetic) regions of interest. Reported data are in g m^−2^

	Area	10 min					20 min					30 min					40 min					50 min				
	m^2^	Min	Max	Median	Mean	SD	Min	Max	Median	Mean	SD	Min	Max	Median	Mean	SD	Min	Max	Median	Mean	SD	Min	Max	Median	Mean	SD
Collar	0.017	3	13	8	8	5	13	133	35	60	64	13	144	75	77	65	42	157	90	96	58	36	152	97	95	58
Shoulders	0.032	0	11	3	5	6	1	22	2	9	12	0	43	4	16	24	12	60	50	41	25	11	73	28	38	32
Sleeves front	0.030	0	14	9	7	7	7	21	8	12	8	3	20	6	10	9	6	35	17	20	15	2	52	17	24	26
Chest medial	0.053	1	7	5	4	3	9	99	20	42	50	6	105	37	49	51	17	141	58	72	63	17	134	72	74	59
Chest lateral	0.040	0	12	6	6	6	1	36	6	14	19	1	42	2	15	23	4	99	13	39	52	12	111	13	45	57
Abdomen medial	0.043	0	17	3	7	9	0	6	3	3	3	0	2	1	1	1	4	99	6	36	54	3	147	6	52	82
Abdomen lateral	0.029	0	10	0	3	*6*	0	3	1	1	1	2	14	10	9	6	0	45	2	16	25	0	61	8	23	33
Low end front	0.062	0	5	3	3	3	0	3	1	1	1	0	2	1	1	1	0	3	3	*2*	2	0	33	3	12	18
Upper back	0.067	0	14	7	7	7	30	52	33	38	12	39	64	56	53	12	61	101	84	82	20	80	117	91	96	19
Sleeve back	0.037	3	24	17	15	11	9	33	22	21	12	14	39	17	23	13	24	53	41	39	15	20	58	51	43	20
Medial mid-back	0.048	0	20	7	9	0	36	73	40	50	0	24	104	39	56	0	49	115	95	86	0	69	124	118	104	0
Lateral mid-back	0.043	0	15	11	8	7	5	24	12	14	9	8	55	15	26	26	20	76	42	46	28	20	87	69	58	35
Medial lower-back	0.036	0	5	4	3	3	13	19	14	15	3	5	41	*9*	18	20	21	70	44	45	25	29	91	73	64	32
Lateral lower-back	0.032	1	11	3	5	6	0	9	1	3	5	1	13	^7^	7	6	12	33	17	21	11	0	35	20	18	17
Low end back	0.061	0	0	0	0	0	0	10	0	3	*6*	0	2	1	1	1	0	0	0	0	0	0	70	0	23	40

T−shirt local sweat absorption data of 4 male athletes are reported for 10, 20, 30, 40 and 50 min run durations, minimum (min) and maximum (max) values, along with median, mean and standard deviation (SD) is reported for each region of interest

## Discussion

The present investigation aimed to study the migration of sweat from the skin to clothing, specifically in cotton and synthetic upper body garments, over 50 min of running exercise performed by male athletes. Based on the obtained values, a series of maps of sweat absorption in such garments were created. Adding to the sweat distribution maps produced in our laboratory (Havenith et al. 2008; Smith and Havenith 2011, 2012), this study provides for the first time quantitative data on total as well as regional absorption of this sweat in the studied garments and clearly demonstrated considerable variation between different garment zones, related to sweat generation in the underlying skin, as well as local fit and contact of the garment.

Thermophysiological responses of core temperature, heart rate and whole body sweat production increased over time (running duration; Figs. 3, 4). The mean rate of core temperature increase was 0.04 °C min^−1^. Whole body sweat production was measured in 10 different running trials and mean sweat rate corresponded to 11.7 g m^−2^ min^−1^. In Fig. 5, it can be observed that whole body sweat production (GSL) linearly increased as function of exercise duration (*r*^2^ = 0.99). On the other hand, a different trend of core temperature development in relation to exercise duration (time) can be observed in Fig. 4, this being non-linear. This suggests that changes in whole body sweat production during exercise are not solely and/or mainly affected by changes in body core temperature. In fact, sweat rate responses during exercise in humans are driven by thermal factors, such as core and skin temperature, but also by non-thermal factors (muscle mechanoreflex, muscle metaboreflex, baroreflex, osmoreflex and chemoreflex) (Crandall et al. 1998; Shibasaki et al. 2003; Kondo et al. 2010). In this regard, it has been suggested that for a given core temperature (thermal input), sweat rate is higher than at rest (i.e., passive heating) because of the contribution of non-thermal factors which, in addition to thermal factors would increase sweat rate (Kondo et al. 2010). The fact that in the current study changes in core temperature during exercise were not related to changes in total body sweat rate confirms that non-thermal factors (i.e., activation of muscle metaboreceptors and mechanoreceptors) highly influence sweating responses in addition to thermal factors (in this case increases in core temperature). The contribution of non-thermal input to whole body sweat production can be also linked to the group of participants involved in the current investigation, these including long-distance runners. In fact, it has been demonstrated that local sweat rate mediated by non-thermal factors (activation of muscle metaboreceptors) is higher in distance runners as compared to sprinters and untrained males (Amano et al. 2011).

T-shirt absorption increases significantly with exercise duration, but starts to plateau after 35 min (Figs. 6, 7), although this was not accompanied by maximal T-shirt moisture saturation. In fact, after 50 min of running exercise, the cotton and synthetic garments reached, on average, only 41 and 18% of the maximal absorption capacity, respectively. The highest local moisture saturation was achieved in the mid-medial back and it was around 56 and 35% in the cotton and synthetic garment, respectively. From the early stages of the running activity (after 15 min), a clear pattern in local sweat accumulation was observed. The maps in Figs. 6 and 7 highlight a decrease in sweat content from medial to lateral and from the top to the bottom, both for front and back of the T-shirt. These patterns were maintained throughout the rest of the running exercise (35–50 min). In line with regional sweat rate data in males (Smith and Havenith 2011), T-shirt sweat accumulation values tend to be higher at the posterior compared to the anterior part of the T-shirt. Overall, local sweat absorption patterns reflect body regional sweat rate as determined by Havenith et al. (2008) and Smith et al. (2011, 2012), although, as expected, values are substantially lower in absolute terms. Body sweat maps were produced with the aim of quantifying regional differences in sweat rate across the body. In these studies, a highly absorbent material was placed directly in contact with the skin, for a 5 min period, to allow collection of local body sweat and the absorbed material was covered by an impermeable film to prevent sweat evaporation during the collection period. In the current study, the sweat produced in the upper body could evaporate or be ventilated directly from the skin but also from the T-shirt. Moreover, to simulate real-life conditions, sweat from the head, forehead, and face was allowed to drip on the garment. As such, the combination of these factors is represented in the data, reflecting realistic wear conditions occurring during exercise.

The inter-regional differences in T-shirt sweat accumulation can be explained by the interactions of physiological, anatomical and clothing factors. When wearing a T-shirt with a regular fit, the top parts, covering chest as well as upper and mid-back, are directly in contact with the body, due to the absence of an air gap. Consequently, these upper T-shirt parts will be directly and constantly in contact with the skin, this facilitating sweat absorption. In addition to this, regional sweat rate distribution, in male runners (Havenith et al. 2008; Smith and Havenith 2011, 2012), shows a consistent pattern of sweat rate reduction from high to low body regions, with the posterior torso, especially at the spine, having the highest values. Therefore the combination of clothing factors, i.e., high fabric-to-skin contact and physiological factors, i.e., high sweat rate, explains the highest sweat accumulation in the top-posterior and top-anterior regions of the T-shirt. Specifically, the medial-upper portion of the T-shirt, in contact with mid-back, upper back and medial chest, showed the highest sweat accumulation.

On the other hand, the bottom parts of a T-shirt (presenting a regular fit) typically hang loose in those regions covering lower back and abdomen, due to specific body shapes (i.e., lumbar curvature) and draping behaviour of clothing. This is likely to result in a relatively large air gap between the T-shirt and the body (Psikuta et al. 2012; Frackiewicz-Kaczmarek et al. 2015), causing a less direct and only intermittent T-shirt-to-skin contact, mainly occurring from air and body movement. This will hamper garment sweat absorption, despite a physiologically high sweat rate at the lower back (Havenith et al. 2008; Smith and Havenith 2011, 2012). In line with this, it can be observed, from the current T-shirt sweat maps that in the bottom posterior parts of the T-shirts sweat accumulation is substantially lower or appears later compared to the top ones, in both cotton and synthetic garments. In particular, the lower-posterior portion of the T-shirt, covering the lower back, starts to show a substantially high sweat content, only after 35 min minutes. Therefore, it can be speculated that most of the sweat accumulated in the low-posterior part of the T-shirt is the result of sweat migration from the top to the bottom T-shirt regions, whereas in these inferior zones the high local sweat rate (Smith and Havenith 2011, 2012) plays a minor role. The same principle applies for the bottom-anterior parts of the T-shirt, covering the abdomen. In fact, the latter regions present a small contact area with the skin and some of these are mostly in contact with the shorts. Additionally, lower abdominal regions present a substantially lower sweat rate, compared to the back (Smith and Havenith 2011, 2012). This may explain the significantly lower sweat content in the T-shirt regions covering the abdomen, in particular the lateral abdomen and the lower hem of the T-shirt at the front, but also at the back. Finally, the low sweat accumulation, being dependent mostly on sweat migration from the superior T-shirt parts, may also contribute to the larger variations in sweat content in the inferior compared to the superior T-shirt zones.

A large part of the individual variation in the total amount of sweat absorbed by the T-shirt was due to the large individual variation in whole body sweat production. In fact, although in the current study it was not possible to simultaneously measure the amount of sweat solely produced in the body parts covered by the T-shirts, using individual GSL as covariant resulted in a 6% lower variance between individuals.

As expected, sweat absorption data were substantially lower in the synthetic garment as compared to the cotton garment; nevertheless, the patterns of sweat distribution appeared to be very similar (Figs. 6, 7). These findings nicely demonstrate that differences in textile properties can determine the absolute amount of sweat absorbed and distributed across the garment, thereby affecting post-exercise body cooling provided, this being approximately 255 and 104 W in the cotton and synthetic garment, respectively [assuming a post-exercise time of 20 min and when sweat evaporation occurs from the skin (Havenith et al. 2013)]. On the other hand, garment fit mainly affects the patterns of sweat absorption distribution in clothing, through its effect on fabric-to-skin contact and air gap thickness. Together with textile properties, environmental factors, such as relative humidity, temperature and air flow, could also influence absolute sweat absorption data.

## Conclusion

This study provided data on sweat accumulation in cotton and synthetic garments occurring during exercise performed by male runners. A clear pattern of sweat absorption reduction from the top to the bottom and from the centre to the sides of the T-shirt was observed in both cotton and synthetic garments. The current results represent useful guidelines for clothing developers when designing products with efficient moisture management features. Given that the sides of the T-shirt contain a significantly lower amount of sweat compared to the central parts, innovative fibre and textile structures should be placed to direct sweat migration from the centre towards the less saturated side regions. The latter would improve sweat management and evaporation, ultimately reducing thermal and sensorial discomfort during exercise as well as heat strain. These data can also be applied as reference values for test methods and apparatus that measure fabric and clothing moisture-related properties. Finally, the large variation in total and local sweat absorption data is a clear sign that clothing customization is required to suit individual body sweat responses. In fact, the design of a single T-shirt, based on mean sweat absorption data may not accommodate the needs of athletes or consumers with extremely low or high sweating responses. Future studies should investigate sweat absorption patterns in a multilayer clothing system as well as in the bra worn in combination with a base layer by female participants. Finally, future investigations should consider changes in garment sweat absorption values in relation to ambient temperature, relative, humidity and wind speed.

## Electronic supplementary material

Below is the link to the electronic supplementary material.

Supplementary material 1 (DOCX 239 KB)

## Abbreviations

ABS_local_: Local sweat absorption
ANOVA: Analysis of variance
ABS_total_: Total sweat absorption
GSL: Gross sweat loss
HR: Heart rate
*T*_core_: Core temperature
*V*O_2max_: Maximum oxygen uptake
