# Supplementary material for: Spatial and temporal migration of sweat: from skin to clothing
Source: Eur J Appl Physiol. 2018 Jul 19;118(10):2155–69. doi: 10.1007/s00421-018-3941-9 (PMC6153606; doi:10.1007/s00421-018-3941-9)
Supplement: Supplementary file 1 — Supplementary material 1 (DOCX 239 KB) [file 421_2018_3941_MOESM1_ESM.docx]

Supplemental digital content 1 Significance levels of comparison of local sweat absorption data after 10 MIN of running


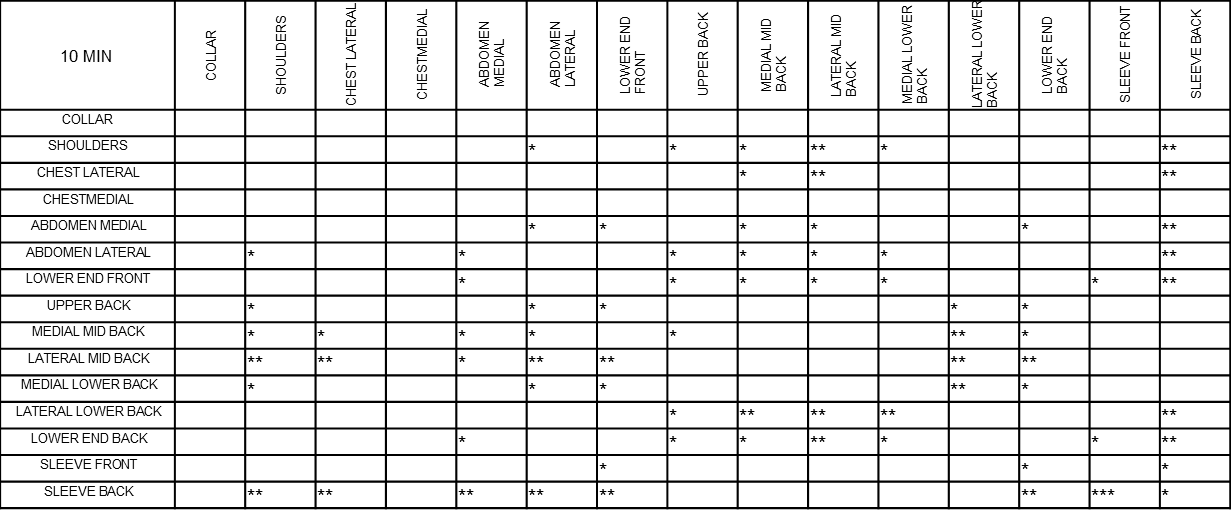


^Data uncorrected for multiple comparisons: p ≤ 0.05 * p ≤ 0.01 ** p ≤ 0.001 ***^

^Data corrected (Bonferroni) for multiple comparisons: p ≤ 0.05 † p ≤ 0.01 †† p ≤ 0.001 †††^

Supplemental digital content 2 Significance levels of comparison of local sweat absorption data after 20 MIN of running


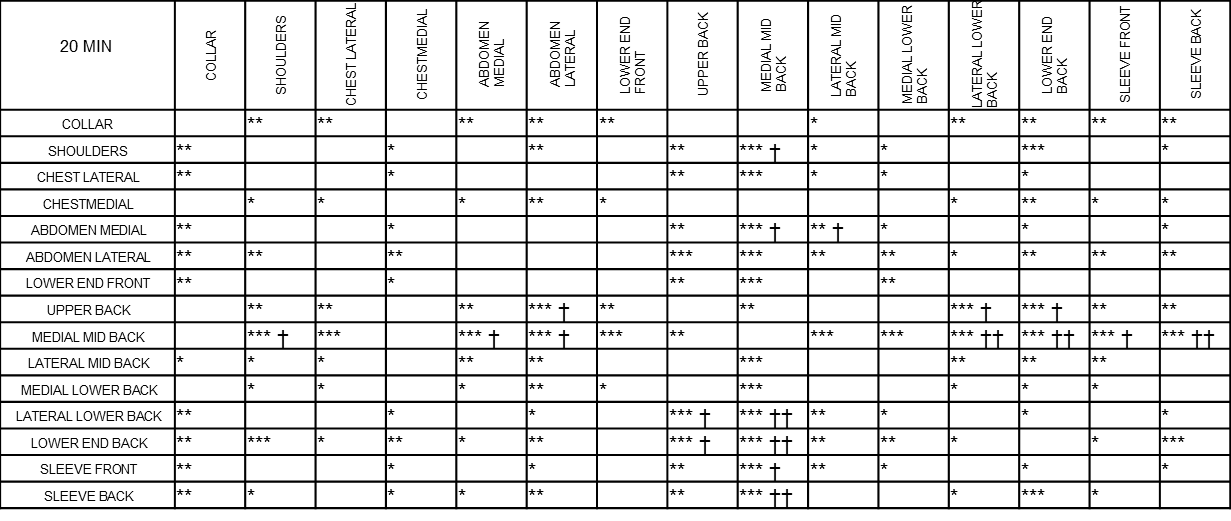


^Data uncorrected for multiple comparisons: p ≤ 0.05 * p ≤ 0.01 ** p ≤ 0.001 ***^

^Data corrected (Bonferroni) for multiple comparisons: p ≤ 0.05 † p ≤ 0.01 †† p ≤ 0.001 †††^

Supplemental digital content 3 Significance levels of comparison of local sweat absorption data after 30 MIN of running


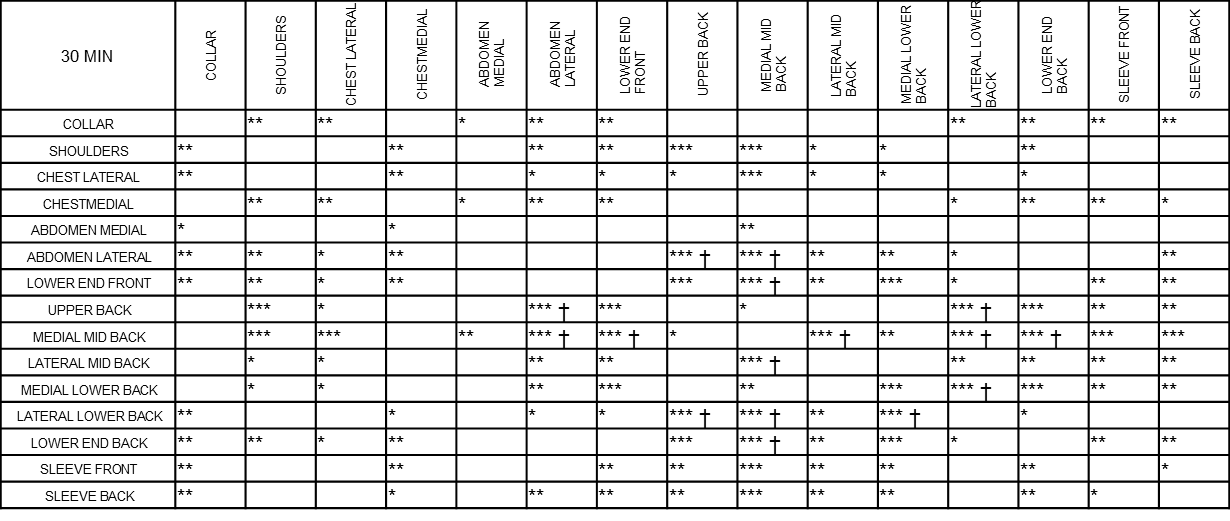


^Data uncorrected for multiple comparisons: p ≤ 0.05 * p ≤ 0.01 ** p ≤ 0.001 ***^

^Data corrected (Bonferroni) for multiple comparisons: p ≤ 0.05 † p ≤ 0.01 †† p ≤ 0.001 †††^

Supplemental digital content 4 Significance levels of comparison of local sweat absorption data after 40 MIN of running


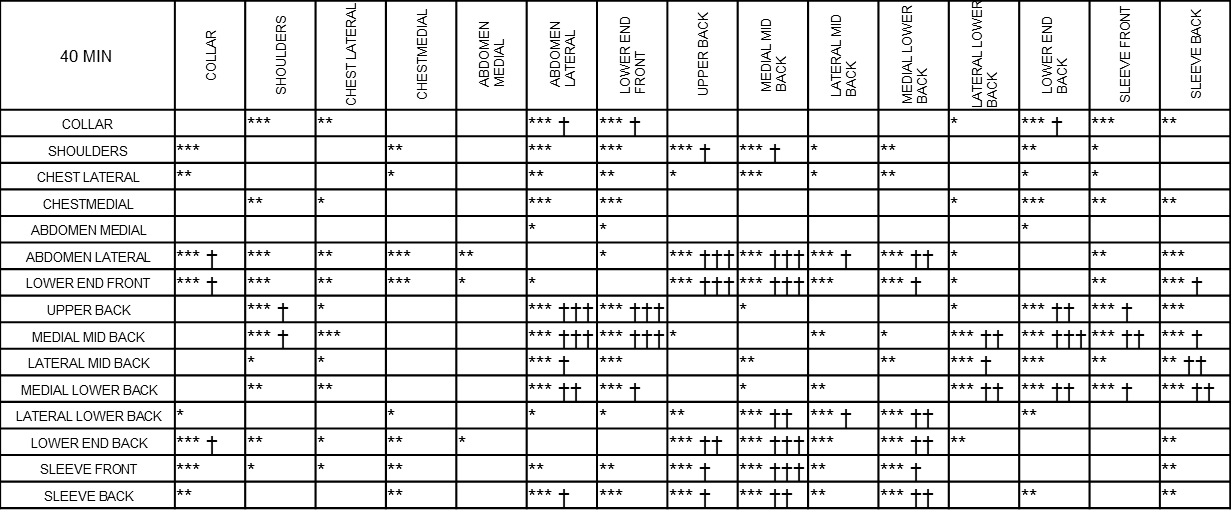


^Data uncorrected for multiple comparisons: p ≤ 0.05 * p ≤ 0.01 ** p ≤ 0.001 ***^

^Data corrected (Bonferroni) for multiple comparisons: p ≤ 0.05 † p ≤ 0.01 †† p ≤ 0.001 †††^

Supplemental digital content 5 Significance levels of comparison of local sweat absorption data after 50 MIN of running


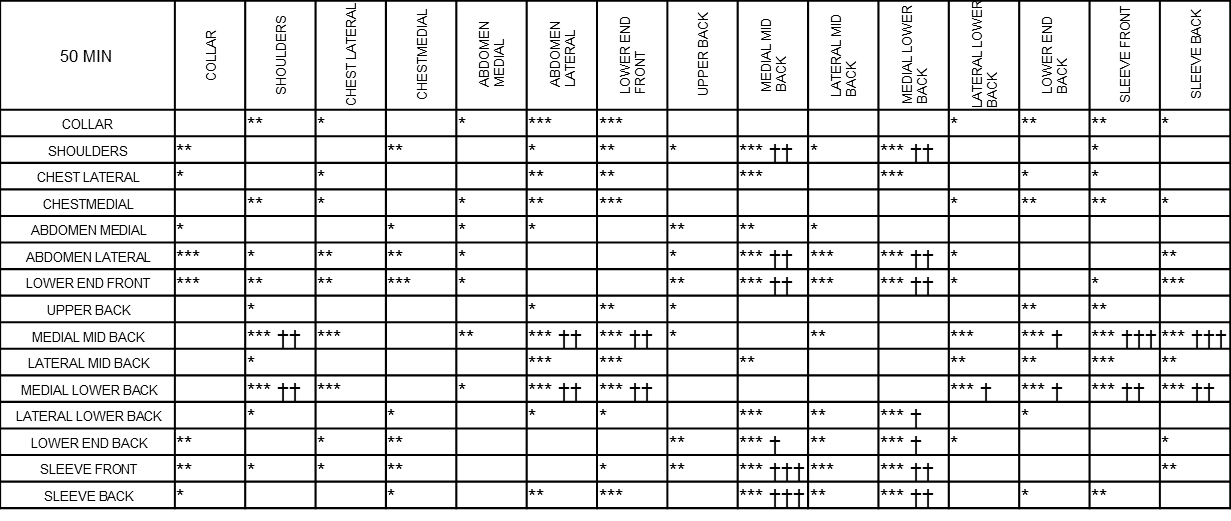


^Data uncorrected for multiple comparisons: p ≤ 0.05 * p ≤ 0.01 ** p ≤ 0.001 ***^

^Data corrected (Bonferroni) for multiple comparisons: p ≤ 0.05 † p ≤ 0.01 †† p ≤ 0.001 †††^
